# Pelvic lipomatosis: US and CT diagnosis

**DOI:** 10.2349/biij.8.2.e12

**Published:** 2012-04-01

**Authors:** Y Yesilkaya, M Duymus, M Topcuoglu

**Affiliations:** 1 Department of Radiology, Hacettepe Hospital, Ankara, Turkey.; 2 Department of Radiology, Kars Kafkas Hospital, Ankara, Turkey.

Dear Editor,

Pelvic lipomatosis is an uncommon benign disease that causes different symptoms due to the compression of pelvic organs by an intrapelvic overgrowth of mature fatty tissue. Engels first described the condition in 1959 [1].

The aetiology of the disease has not been established. However, it has been speculated that the fat proliferation might be associated with chronic pelvic inflammation due to chronic urinary tract infection [2]. In adults, endocrine diseases such as Cushing’s syndrome, hypothyroidism, insulin-secreting tumours, and neoplastic disease involving the hypothalamus (mainly craniopharyngioma) may also be a factor in the acquired obesity [2, 3].

Imaging is crucial in the diagnosis of pelvic lipomatosis. CT scans are considered to be the most effective and essential form of examination. The technique ensures dependable diagnosis of the disease principally because the absorption coefficient of the intrapelvic fatty tissue calculated by computer is distinct from that of other tissues.

In the case we describe here, the diseases above could be ruled out based on the pattern of fat distribution, the clinical situation, and laboratory data. Laboratory results were all within normal limits. Pelvic lipomatosis typically exhibits a wide variety of symptoms including lumbago, discomfort of the lower abdomen, low-grade fever, recurrent urinary infections, frequent urination, dysuria, constipation, and hypertension [2, 4]. Our patient only had non-specific back pain.

**Figure 1:**
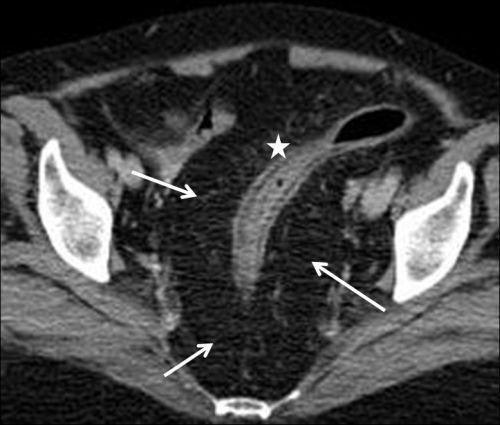
Pelvic computed tomography (CT) scan showing compression of the sigmoid colon (star) by an unencapsulated, homogenous soft tissue mass (white arrows).

**Figure 2:**
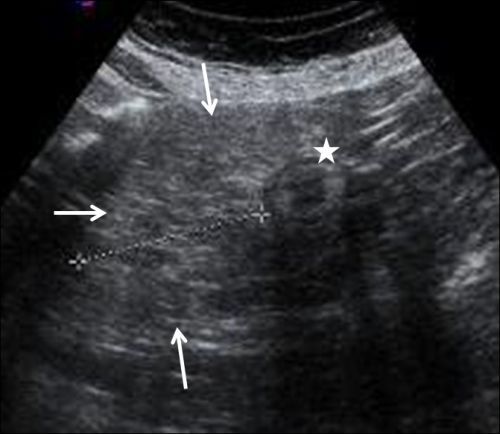
Pelvic ultrasound examination showing hyperechoic fat tissue (white arrows) surrounding the sigmoid colon (star).

Pelvic computed tomography (CT) examination revealed compression of the sigmoid colon by an unencapsulated, homogeneous soft tissue mass with the same attenuation as normal subcutaneous fat. Superior displacement and narrowing of the sigmoid was noted (Figure 1). No enhancement was noted after administration of the contrast agent. No evidence of any other soft tissue mass was present, and all the tissue planes were preserved. Pelvic ultrasound (US) examination revealed hyperechoic fat tissue surrounding the sigmoid colon (Figure 2).

The combination of US and CT findings was successful in suggesting a diagnosis of pelvic lipomatosis. Such radiographic and physical findings are often sufficiently definitive that surgery is not indicated. The ability to make a specific diagnosis of pelvic lipomatosis without an invasive procedure is quite helpful [5, 6]. It is therefore important to note that the CT scans of our patient only showed specific findings, namely increased fat as the only abnormality, in the absence of other mass lesion.
